# The effect of mindfulness training on resilience and decision-making strategy in women with unwanted pregnancy (a quasi-experimental study)

**DOI:** 10.1186/s40359-025-03864-w

**Published:** 2025-12-23

**Authors:** Zahra Alikhani Faradonbe, Monireh Toosi, Parvin Ghaemmaghami, Azar Nematollahi

**Affiliations:** 1 Student Research Committee, School of Nursing and Midwifery, Shiraz University of Medical Sciences, Shiraz, Iran; 2 Community Based Psychiatric Care Research Center, School of Nursing and Midwifery, Shiraz University of Medical Sciences, Shiraz, Iran; 3 Biostatistics PhD, School of nursing and Midwifery, Shiraz university of Medical Sciences, Shiraz, Iran; 4 Department of Midwifery, School of nursing and Midwifery, Shiraz university of Medical Sciences, Shiraz, Iran

**Keywords:** Mindfulness, Resilience, Decision-Making style and strategy, Unwanted pregnancy

## Abstract

**Background:**

Unplanned pregnancy, as a stressful event, affects women`s mental health and leads to an increase in abortion attempts. This study was conducted to determine the effect of mindfulness training on resilience and decision-making strategy in women with unwanted pregnancy.

**Method:**

The present study is a quasi-experimental study. Eligible samples were randomly divided into two groups: experimental (*n* = 32) and control groups (*n* = 32) after selection from 6 comprehensive health centers in Shiraz. Data collection tools included a questionnaire on demographic and fertility characteristics, a questionnaire on women’s decision-making strategies regarding abortion or continuation of unwanted pregnancy, and the Connor-Davidson Resilience Scale. After the pre-test, the mindfulness technique training program was administered to the experimental group via mobile application designed by researcher. The control group received routine care. Independent t-test, paired t-tests and Pearson correlation, were used to analyze the data with SPSS version 25 and significance level was set at *P* < 0.05.

**Results:**

The results showed that there was no significant difference between the mean score of resilience (intervention: 44.28 ± 8.45, control: 43.68 ± 8.26), dimensions of resilience, mean score of decision-making (intervention: 68.25 ± 10.28, control: 69.31 ± 10.51,), and dimensions of decision-making before the intervention between the two groups, but after the intervention, this difference became significant in all the mentioned variables: mean scores of resilience (intervention: 59.78 ± 9.16, control: 42.15 ± 6.34, *p* < 0.001) and mean scores of decision-making (intervention: 54.06 ± 8.47, control: 70.21 ± 10.64, *p* < 0.001). Also, a significant inverse relationship was observed between the score of resilience and the score of decision-making (*p* < 0.001), in a way that with increasing resilience (mean difference in intervention:15.5 ± 3.9), the decision-making for abortion decreased (mean difference in intervention: -14.18 ± 4.86).

**Conclusion:**

Utilizing a mindfulness training strategy for couples with unintended pregnancies who are not inclined to continue the pregnancy can have a positive effect on their decision-making. Therefore, conducting this study in different communities with varying cultures and customs, as well as larger samples in future studies for a better comparison of the results obtained is recommended.

## Introduction

Unwanted pregnancy is a global problem that affects the lives of women, their families, and ultimately society [1]. This crisis can affect women’s psychological well-being and leading to an increased likelihood of abortion or negative and harmful effects on the fetus [2]. Today, the rate of unwanted pregnancy is recognized as one of the health indicators of society and has led to an increased prevalence of induced abortion. Statistics show that about 40% of pregnancies worldwide are unwanted, and ultimately, 50% of these pregnancies result in induced abortion, while %38 lead to the birth of an unwanted child. In both scenarios, the health of mothers and children is put at risk [3]. Furthermore, the prevalence of unintended pregnancy in Iran has been reported to be % 30.6 [4].

On the other hand, in recent years, the fertility trend in Iran has been declining [5]. Results from available statistics in Iran indicate that the total fertility rate has decreased from approximately 7.7 children per woman in 1966 to 2.17 in 2000 and further to 1.65 children in 2021 [6–9]. Following this decline in fertility in Iran, policies to encourage childbearing were considered in the 2010s, and finally in November 2021, the Law on Family and Youth Protection was promulgated [10]. In line with the implementation of population policies, maintaining and promoting the health of individuals in society, especially planning for safe pregnancies in order to protect the health of mothers and children is a priority [11]. Indeed, women need to be prepared for a safe pregnancy and childbirth, as well as for emotional bonding and newborn care [12]. Pregnancy is a stage for accepting new roles and responsibilities [13]. Meanwhile, the characteristic of unwanted pregnancy is such that it challenges the process of acceptance and adaptation to it [14].Women consider unwanted pregnancy as a threatening and challenging situation [15, 16]. Unintended pregnancy is associated with fear, worry, and anxiety during pregnancy [12]. During pregnancy, focusing on the mind (mindfulness) is proposed as an effective strategy for managing anxiety. In fact, mindfulness-based interventions can contribute to the mental well-being and empowerment of pregnant women and have a positive impact on the reproductive process [17]. Mindfulness helps individuals to identify situations that cause anxiety and stress, gain a better understanding of themselves and recognize their strengths and weaknesses, and then learn coping strategies to deal these situations and increase their level of resilience [18].

Individuals who practice mindfulness techniques experience a greater sense of flexibility and are guided away from the challenges of thoughts and problems towards greater calmness and resilience. When faced with unpleasant and difficult situations, they try to act wisely instead of reacting impulsively [19]. It seems that mindfulness significantly contribute to improving quality of life and develop positive skills such as resilience. In fact, one of the functional mechanisms of mindfulness is its effect on increasing the level of resilience of individuals [20]. Resilience is the ability of an individual to maintain a biological and psychological balance in dangerous situations [21]. Indeed, resilience is a phenomenon that results from natural human adaptive responses and empowers individuals to achieve success and overcome threats, despite facing serious challenges. Resilience is the return to initial equilibrium or reaching a higher level of equilibrium in threatening situations and thus enables facing a problem such as an unwanted pregnancy [22]. In this regard, the results of a study conducted in Iran in 2020 showed that group mindfulness training is effective in improving resilience and emotion regulation strategies in pregnant women. In fact, in mindfulness sessions, pregnant women were able to discuss effective coping strategies with stress and learn more practical ways; therefore they were in better psychological and emotional conditions and achieved a higher level of resilience [23]. Furthermore, according to evidence, one of the most effective methods for intervening in anxiety control and increasing resilience is the mindfulness therapeutic approach [24].

Considering the effective role of midwives in prenatal care, such as counseling and increasing the level of awareness of pregnant women, it seems that mindfulness training can reduce anxiety, stress, and increase the resilience of pregnant women. Mindfulness can also help women cope with the psychological burden of unwanted pregnancy and support informed decision-making. Since such research has not been conducted in Iran in previous studies, this study was conducted to determine the effect of mindfulness training on resilience and decision-making in women with unwanted pregnancies.

## Methods

The present study is a quasi-experimental design with a pre-test, post-test, and control group. The sample size was determined using G*Power 3 software, considering a type I error rate (α) of 0.05, a type II error rate (β) of 0.20 (resulting in a 95% confidence level and 80% power), based on data from a similar thesis [25] (Cohen’s d effect size = 0.62 and f effect size = 0.31), and a correlation of 0.3 between measurement times. This calculation yielded a total sample size of 56 participants. To account for an anticipated attrition rate of at least 10%, the total sample size was increased to 64 participants; finally, data analysis was conducted on 32 participants in each group.

The statistical population of this research consisted of 64 women who, along with their partners, were not inclined to continue their pregnancy, were less than 20 weeks pregnant, and had visited healthcare centers in Shiraz during the year 2024. The inclusion criteria for the study were: voluntary participation of individuals with unintended pregnancies, having a high school diploma or higher education, not currently using psychotropic medication or receiving other psychological services, gestational age of less than 20 weeks, lack of physical illness or mental disorder, living with their spouse, and lack of an unintended pregnancy that met the criteria for therapeutic abortion. The exclusion criteria included: non-response to telephone follow-ups for assessing learning of online training, irregular completion of taught exercises and interventions, unwillingness to continue participation in the research after enrollment or during follow-up, incomplete completion of questionnaires, and contracting an illness during the intervention period.

In this research, after obtaining permission from the Ethics Committee of Shiraz University of Medical Sciences with the code IR.SUMS.NUMIMG.REC.1402.060 and receiving a letter of introduction from the School of Nursing and Midwifery, the researcher referred to healthcare centers in Shiraz for sampling. The sampling method was a cluster random sampling. First, 6 regions (cluster) were randomly selected from 11 regions of Shiraz city. Then, from each cluster, one comprehensive health center was selected for sampling. Finally, 6 centers were selected for sampling. After identifying the health centers and determining the participants who met the inclusion criteria, the objectives and procedure were explained to them. Out of the 86 introduced individuals, only 70 women met the inclusion criteria. They were randomly assigned to the control group and the intervention group in R software. In fact, the zero codes were given to the control group, whereas the one codes were given to the intervention group (35 participants in each group).

At the beginning of the study, written informed consent was obtained from all participants, and then the demographic questionnaire, the Connor-Davidson Resilience Scale, and the questionnaire on women’s decision-making strategies regarding abortion or continuation of unintended pregnancy were completed in both groups. Following the pre-test, the mindfulness technique training program was administered to the intervention group via a mobile application according to Table 1. This application was designed to be installed on Android mobile phones.

**Table 1:** Summary of mindfulness skills training sessions according to Kabat-Zinn, Reibel, & Santorelli (2010**)**

Session	Content
1	Presentation of goals and expectations, pre-test, introduction to mindfulness as a way of life, eating exercise, feedback and discussion about eating exercise, body scan, feedback and discussion about body scan
2	Yoga, discussion about the interaction between mindful activities, introduction to homework and body scan, discussion about the attitude of mindfulness (non-striving and non-rumination), introduction to sitting meditation, feedback and discussion about sitting meditation
3	Yoga, sitting meditation, seeing and hearing exercises, discussion about the attitude of mindfulness (non-judging, patience), walking meditation, mindfulness of breathing, sitting meditation as practice, dealing with thoughts by bringing them under control, generalization of the 3-minute breathing space exercise
4	Yoga, sitting meditation, awareness of breath, body awareness, sounds, thoughts, discussion about the attitude of mindfulness (acceptance and letting go), addressing difficult feelings and emotions
5	Yoga, sitting meditation, awareness of breath, body awareness, sounds and thoughts, introduction of problematic thoughts and dangers, breathing exercises for anger management, mindfulness and communication
6	Yoga, sitting meditation, mindfulness and compassion meditation, conclusion, discussion on how to integrate mindfulness practices into one’s lifestyle, post-test

The training was delivered to each individual in the intervention group through an interactive multimedia mobile application that included question-and-answer sessions with patients and addressed potential queries. It is important to note that during the initial in-person meeting, the application was installed on the mobile phones of all participants, and its functionality and usage were explained to them.

The application consisted of a (main) content training section and (secondary) sections for information, entertainment, and music to enhance user engagement. The software was designed such that one training session was made available to participants each week (total of 6 sessions), and after completing the exercises for each session, access to the educational content and exercises for subsequent sessions was granted. The content of the educational intervention was developed based on mindfulness skills training sessions according to Kabat-Zinn, Stahl, Goldstein, and Santorelli (2010). One week after the intervention, the Connor-Davidson Resilience Scale and the questionnaire on women’s decision-making styles and strategies regarding abortion or continuation of unintended pregnancy were completed again by women with unintended pregnancies. Furthermore, at the end of the intervention, the application was provided to the control group to ensure educational equity, and finally, the obtained statistical data were interpreted.

The data collection instruments in this study included three questionnaires: the demographic and fertility questionnaire, the Connor-Davidson Resilience Scale, and the questionnaire on women’s decision-making styles and strategies regarding abortion or continuation of unintended pregnancy.

### Demographic and fertility questionnaire

This Questionnaire included 18 questions: contact phone number, date of last menstrual period (to determine gestational age), number of pregnancies and abortions, age, occupation before and during pregnancy, level of education.

###  Connor-Davidson resilience scale

This scale was developed in 2003 through a review of research literature from 1979–1991 in the field of resilience. It consists of 25 items that correspond to classified characteristics of resilience. The scoring method for this questionnaire is based on a five-point Likert scale, where responses ranging from ‘completely untrue’ to ‘always true’ are scored, 1, 2, 3, 4, 5,respectively. The minimum score of the questionnaire is 25 and the maximum is 125. This test has 5 factors: perceived personal competence, trust in one’s own instincts - tolerance of negative affect, positive acceptance of change and secure relationships, self-control, and spiritual influences. The developers of this scale believe that this questionnaire is well-suited to differentiate resilient individuals from non-resilient individuals in both clinical and non-clinical groups and can be used in research and clinical settings [21]. Mohammadi adapted it for use in Iran and reported its reliability with a Cronbach’s alpha of 0.93. Furthermore, in their research, the questionnaire’s validity was confirmed by experts [26].

### Questionnaire on women’s decision-making strategies regarding abortion or continuation of unwanted pregnancy

This questionnaire consists of 27 items that assess 4 construct factors: resistance to acceptance, avoidant-justificatory strategies, analytical strategies, and confirmatory strategies. Items 1, 2, 3, 4, 5, 6, 7, 8, 9, 10, 11, 12, 13, 14, 15, 19, 20, 21, 22, and 23 are in a five-point Likert scale format, where ‘not option’ is scored 0, ‘not at all’ is scored 1, ‘a little’ is scored 2, ‘moderate’ is scored 3, and ‘a lot’ is scored 4. The 4 items (16, 17, 18, and 24) are negatively worded and reverse-scored. For items 25, 26, and 27, a ‘yes’ response is scored 2, and a ‘no’ response is scored 1. The minimum score of the questionnaire is 27 and the maximum is 108. A higher score indicates an increase in the decision to have abortion.

In the study by Roqieh Norizadeh and colleagues, the Cronbach’s alpha Coefficient of the scale was calculated as 86%, and the instrument’s stability was assessed using the Pearson correlation coefficient, yielding 92%. Additionally, the Interclass Correlation Coefficient (ICC) for the designed instrument across two measurements taken two weeks apart was found to be 0.94, indicating high stability of the instrument [27].

In this study, the mean and standard deviation was used to describe quantitative data, and frequency (percentage) was used to describe qualitative data. The correlation between quantitative variables was assessed using the Pearson correlation test (non-parametric equivalent: Spearman), and the relationship between qualitative variables was evaluated using the chi-square test. To investigate and evaluate the main objective, the independent samples t-test was used to compare the mean and changes in the mean resilience score between the two groups before and after the intervention. Additionally, the paired samples t-test was used to compare the within-group means before and after the intervention. The normality of the data was assessed using the Shapiro-Wilk test, and parametric tests were used in this study. All statistical analyses were performed using SPSS version 25, and the significance level was set at *p* < 0.05.

## Results

**Fig. 1:**
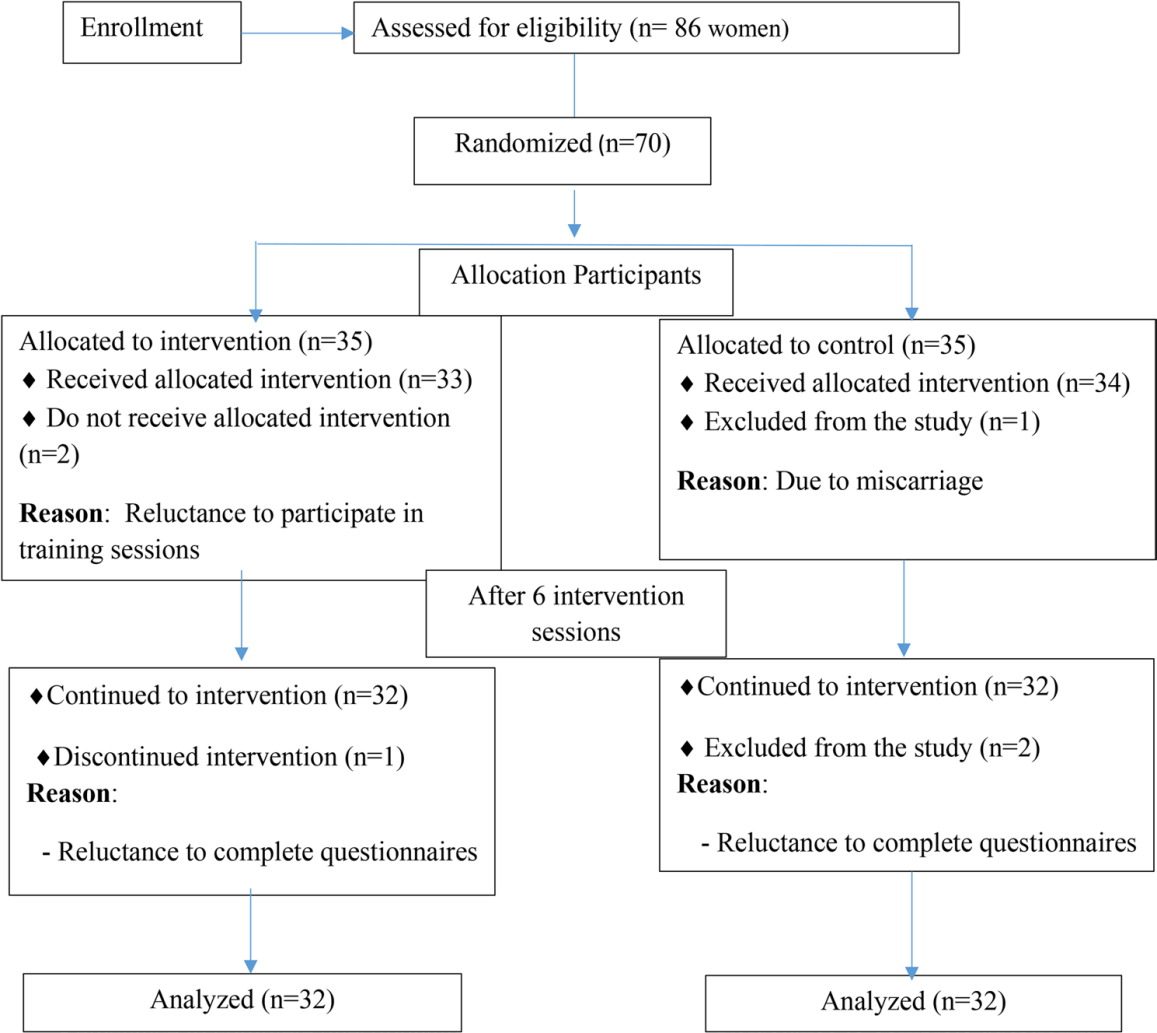
Flow diagram of study design

In this study, after checking the inclusion criteria, 70 eligible women were equally divided into the study groups including control group (*n* = 35) and intervention group (*n* = 35). In the control group, 3 people (one before the intervention and two after the intervention) and in the intervention group, 3 people (two before the intervention and one after the intervention) were excluded of the study at different stages (Fig. 1). Finally, data from 64 study participants (32 in each group) were included in the analysis.

The evaluation of demographic variables showed that the mean age of participants in the intervention group was 29.84 ± 6.69 years and in the control group was 31.09 ± 7.62 years. The mean age of husbands in the intervention group was 35.09 ± 7.91 years and in the control group was 36.34 ± 8.12 years. Based on the chi-square test, there was no statistically significant difference between the control and intervention groups in terms of age, spouse’s age, education level, husband’s education level, occupation, husband’s occupation, and gestational age (*p* > 0.001) (Table 2).

**Table 2:** Frequency distribution of demographic characteristics of individuals with unintended pregnancy

Variable	Subgroup	Intervention group		Control group	
		number	Percent	number	Percent
Age	15–29	16	50.0	12	37.5
	30–39	14	43.8	15	46.9
	40–49	2	6.3	5	15.6
Husband’s Age	20–29	6	18.8	8	25.0
	30–39	19	59.4	12	37.5
	40–49	5	15.6	10	31.3
	50–59	1	3.1	2	6.3
	60–69	1	3.1	0	0.0
Education	Diploma	13	40.6	14	43.8
	Bachelor’s	16	50.0	15	46.9
	Master’s & PhD	3	9.4	3	9.4
Husband’s Education	Diploma	15	46.9	10	31.3
	Bachelor’s	11	34.4	16	50.0
	Master’s & PhD	6	18.8	6	18.8
Occupation	Housewife	21	65.6	19	59.4
	Employee	11	34.4	13	40.6
Husband’s Occupation	Unemployed	1	3.1	4	12.5
	Employee	13	40.6	15	46.9
	Self-employed	18	56.3	13	40.6
Gestation Age	1–5 weeks	10	31.3	7	21.9
	6–10 weeks	16	50.0	22	68.8
	11–15 weeks	5	15.6	3	9.4
	16–19 weeks	1	3.1	0	0.0

Comparison of the mean scores of resilience dimensions within and between the intervention and control groups before and after the intervention, according to the results of paired and independent t-tests, showed that the two groups did not have a significant difference in any of the resilience dimensions before the intervention. However, after the intervention, the mean scores showed a significant difference in all dimensions (*p* < 0.001). The within-group analysis, a statistically significant difference in the mean scores of all resilience dimensions was observed only in the intervention group after the intervention compared to before it. Furthermore, the comparison of the mean total resilience score and its changes showed that there was no significant difference in the mean resilience score between the two groups before the intervention (*p* = 0.76), but this difference was significant after the intervention (*p* < 0.001). On the other hand, the changes in the mean resilience scores in the intervention group were significant and equal to 15.5 ± 3.9, while in the control group, it was − 1.5 ± 3.38, indicating a significant improvement in resilience in the intervention group compared to the control group (Table 3).

**Table 3:** Comparison of mean scores of resilience and its dimensions

Resilience Dimensions	Time	Intervention group Mean ± SD	Control group Mean ± SD	*p*-value ^a^
Perception of Personal Competence	Pre-test	13.50 ± 2.92	13.87 ± 3.07	0.619
	Post-test	18.65 ± 2.87	12.03 ± 2.45	< 0.001
	*p*-value ^b^	< 0.001	< 0.001	
Trust in Personal Instincts - Tolerance of Negative Affect	Pre-test	12.75 ± 3.00	12.06 ± 3.19	0.378
	Post-test	16.59 ± 3.06	12.65 ± 2.40	< 0.001
	*p*-value ^b^	< 0.001	0.288	
Positive Acceptance - Change and Secure Relationships	Pre-test	8.9 ± 2.93	9.59 ± 2.63	0.328
	Post-test	12.37 ± 2.64	9.56 ± 2.56	< 0/001
	*p*-value ^b^	< 0.001	0.946	
Self-Control	Pre-test	5.09 ± 1.74	4.65 ± 1.49	0.286
	Post-test	7.31 ± 1.53	4.56 ± 1.50	< 0.001
	*p*-value ^b^	< 0.001	0.768	
Spiritual Influences	Pre-test	3.65 ± 1.15	3.46 ± 1.10	0.509
	Post-test	4.56 ± 1.45	3.34 ± 1.09	< 0.001
	*p*-value ^b^	0.004	0.613	
Mean Resilience Score	Pre-test	44.28 ± 8.45	43.68 ± 8.26	0.766
	Post-test	59.78 ± 9.16	42.15 ± 6.34	< 0.001
	Changes	15.5 ± 3.9	-1.5 ± 3.38	< 0.001
	*p*-value ^b^	< 0.001	0.018	

a: Independent –test

b: paired-t test

Comparison of the mean scores of decision-making dimensions within and between the intervention and control groups before and after the intervention, based on the results of paired and independent t-tests, also showed that the two groups did not have a significant difference in any of the decision-making dimensions before the intervention. However, after the intervention, the mean scores showed a significant difference in all dimensions (*p* < 0.001). Furthermore, the comparison of the mean score of women’s decision-making styles and strategies and its changes showed that there was no significant difference in the mean decision-making score between the two groups before the intervention (*p* = 0.693), but this difference was significant after the intervention (*p* < 0.001). On the other hand, the change in the mean decision-making score regarding abortion in the intervention group was − 14.18 ± 4.86, which indicates that the tendency towards abortion decreased (Table 4).

**Table 4:** Comparison of mean scores of Decision-Making and its dimensions

Decision-Making Dimensions	Time	Intervention group Mean ± SD	Control group Mean ± SD	*p*-value ^a^
Avoidance-Justification Strategies	Pre-test	34.15 ± 6.58	35.31 ± 6.27	0.475
	Post-test	25.46 ± 4.53	35.53 ± 6.06	< 0.001
	*p*-value ^b^	< 0.001	0.716	
Resistance to Acceptance	Pre-test	30.09 ± 6.35	30.03 ± 4.46	0.946
	Post-test	23.18 ± 4.33	30.56 ± 4.22	< 0.001
	*p*-value ^b^	< 0.001	0.463	
Analytical Strategies	Pre-test	13.81 ± 3.59	13.21 ± 2.68	0.457
	Post-test	10.46 ± 2.28	13.59 ± 2.98	< 0.001
	*p*-value ^b^	< 0.001	0.526	
Confirmatory Strategies	Pre-test	14.93 ± 1.89	15.00 ± 2.17	0.903
	Post-test	12.81 ± 1.94	14.96 ± 2.48	< 0.001
	*p*-value ^b^	< 0.001	0.930	
Mean Decision-Making Score	Pre-test	68.25 ± 10.28	69.31 ± 10/51	0.693
	Post-test	54.06 ± 8.47	70.21 ± 10.64	< 0.001
	Changes	-14.18 ± 4.86	0.906 ± 9.45	< 0.001
	*p*-value ^b^	0.592	< 0.001	

a: Independent –test

b: paired-t test

Comparison of the correlation between resilience scores and decision-making strategies for abortion scores within and between the intervention and control groups before and after the intervention showed a significant inverse correlation between resilience scores and decision-making scores before and after the test in both the control and intervention groups (*p* < 0.001). This means that with an increase in resilience, the tendency towards abortion decreased (Table 5).

**Table 5:** Results of the relationship between resilience score and Decision-Making strategies score

Group	variables	Before intervention		After intervention	
		Pearson Correlation Coefficient	*p*-value ^a^	Pearson Correlation Coefficient	*p*-value ^a^
Intervention	Resilience with Decision-Making Strategies	-0/819	< 0.001	-0/650	< 0.001
Control	Resilience with Decision-Making Strategies	-0/673	< 0.001	-0.216	< 0.001
Total	Resilience with Decision-Making Strategies	-0/748	< 0.001	-0.793	< 0.001

a: Pearson correlation test

## Discussion

The present quasi-experimental study was conducted in Iran to investigate the effect of mindfulness training on resilience, decision-making strategies for abortion or continuing pregnancy in women with unintended pregnancies. The results of the present study showed that mindfulness increased resilience in women with unintended pregnancies and also had a positive impact to make decision to continue the pregnancy /avoid abortion.

The findings of the present study is consistent with the results of the research by Sarbandi et al. (2015), showed that the mindfulness-based childbirth and parenting program could create a significant difference in the variables of pregnancy anxiety and resilience of couples [28]. Also, the results of the study by Jafari-Shalkohi et al. (2020) showed that mindfulness training intervention had a significant effect on resilience and cognitive strategies for emotion regulation in pregnant women [29]. Furthermore, the results of a meta-analysis by Cuijpers et al. (2023), showed that psychological interventions are effective in treating prenatal depression, with effects lasting at least 6 to 12 months and also impacting social support, anxiety, functional disability, parental stress, and marital stress [30]. Since mindfulness is considered a psychological intervention and unintended pregnancy is associated with depression and anxiety, our study is in line with this study. Nevertheless, no research has directly investigated the impact of this type of intervention on the resilience of women with unintended pregnancies, and the present study provides, for the first time, evidence of the effectiveness of mindfulness training programs for this group of women, highlighting its novelty.

In explaining the present findings, it can be argued that mindfulness training, by strengthening resilience and the ability to tolerate challenges, stress, and anxiety in situations where pregnant women face stressful issues, can protect them against stress-induced mood dysfunction [31].

In the present study, the score for the control dimension increased in the intervention group after the intervention, and pregnant women expressed a greater sense of control after receiving mindfulness training. In fact, during the mindfulness sessions, pregnant women were able to discuss effective stress coping strategies and learn more practical methods.

Furthermore, in this study, the perception of individual competence dimension increased in the intervention group after mindfulness training. Among the constructs present in mindfulness is self-compassion. Through self-compassion training, such as loving-kindness meditation, pregnant women achieve the ability to deal patiently and non-judgmentally with life’s adversities and their own and others’ mistakes. Adopting a compassionate perspective in women with unintended pregnancies leads to an improved perception of their individual competence.

In the present study, mindfulness had a positive impact to make decision to continue the pregnancy/avoid abortion. Consistent with the present study, in the study of Aghamohammadi and Jokar (2024), the results indicated that mindfulness positively predicts rational decision-making among students [32]. Also, the results of the study by Al-Mohammadi (2025) showed that mindfulness was significantly correlated with positive decision-making among teachers [33]. On the other hand, the results of the study by Liu et al. (2018) and the study by Martinovich (2017) also confirmed the relationship between mindfulness and decision-making [34, 35]. It should be noted that these studies were not conducted on women with unintended pregnancies, and the generalizability of the results of these studies to the present study is limited.

Furthermore, in this study, the confirmatory strategies dimension decreased in the intervention group after mindfulness training. The decision regarding abortion or continuation of pregnancy is influenced by resistance to accepting the reality of the pregnancy and avoidance of perceived threats arising from continuing the pregnancy. Since the decision-making process in the face of unexpected pregnancy is constantly based on avoiding threats and harms, the approach and chosen strategy of women will vary depending on the type and intensity of perceived threats.

The timing and stage of pregnancy diagnosis, along with support resources, play a significant role as influential factors in the acceptance and confirmation of decisions. For instance, a late diagnosis of pregnancy after the fetal heartbeat is established can lead to its non-confirmation. Among the components of support resources, spousal support, along with cultural norms and social laws, greatly impacts the confirmation of these decisions. Therefore, the decision-making path in the face of unintended pregnancy is such that women, considering their circumstances and available resources, try to choose a path that involves the least harm and threat and the most acceptance and confirmation.

Furthermore, in this study, the avoidant-justificatory strategies dimension decreased in the intervention group after mindfulness training. In the avoidant decision-making style, the individual avoids making a decision and delays it as much as possible [36].The attention and focus that are examined in mindfulness cause individuals to be present in the current situation and become fully cognitively engaged with the existing problem. Therefore, it is logical that the more mindfulness an individual possesses, the less they will resort to an avoidant style [29].

Our findings showed that there is a significant relationship between resilience and decision-making strategies. Consistent with the present study, the research by Al-Nidani et al. (2024), showed a positive correlation between resilience and decision-making strategies among the students [37]. Also Shin et al. (2015), in a study, found that resilience and decision-making strategies explained 46% of the variance in career decision-making difficulties [38], which is consistent with the results of the present study and suggests that strengthening resilience improves decision-making.

One of the strengths of the present study was that mindfulness was able to help women cope with the psychological burden of unwanted pregnancy and support informed decision-making. Limitations of the study include non-face-to-face training, small sample size, quasi-experimental design, self-report measures, and cultural sensitivity of the topic. The uncertainty of doing the exercises at home was also one of our limitations, and the researcher followed up on the exercises through phone calls. Also, considering that the education was delivered through a mobile application program, by including various sections, efforts were made to make the training attractive to the audience.

## Conclusion

The results of the present study showed that mindfulness increased resilience in women with unintended pregnancies and also had a positive impact to make decision to continue pregnancy/ avoid abortion. Utilizing a mindfulness training strategy for couples with unintended pregnancies who are not inclined to continue the pregnancy can have a positive effect on their decision-making. Therefore, it is suggested that a mindfulness training program for pregnant mothers and their husbands be included in the agenda of health centers. Also, conducting this study in different communities with various cultures and customs, as well as conducting it with larger samples in future studies for a better comparison of the results obtained, is recommended.

## Data Availability

They are available from the corresponding author on reasonable request.
